# U1 small nuclear ribonucleoproteins (snRNPs) aggregate in Alzheimer’s disease due to autosomal dominant genetic mutations and trisomy 21

**DOI:** 10.1186/1750-1326-9-15

**Published:** 2014-04-28

**Authors:** Chadwick M Hales, Nicholas T Seyfried, Eric B Dammer, Duc Duong, Hong Yi, Marla Gearing, Juan C Troncoso, Elliott J Mufson, Madhav Thambisetty, Allan I Levey, James J Lah

**Affiliations:** 1 Department of Neurology, Emory University School of Medicine, Atlanta 30322, Georgia; 2 Center for Neurodegenerative Disease, Emory University, Whitehead Research Building, 615 Michael Street, Room 505C, Atlanta 30322, Georgia; 3 Department of Biochemistry, Emory University School of Medicine, Atlanta 30322, Georgia; 4 Robert P. Apkarian Integrated Electron Microscopy Core, Emory University, Atlanta 30322, Georgia; 5 Department of Pathology, Emory University School of Medicine, Atlanta 30322, Georgia; 6 Departments of Pathology and Neurology, Johns Hopkins School of Medicine, Baltimore, Maryland 21205, USA; 7 Department of Neurological Sciences, Rush University, Chicago, IL 60612, USA; 8 National Institute of Aging, National Institute of Health, Bethesda, Maryland 20892, USA

**Keywords:** Spliceosome, snRNP, Alzheimer’s disease, Down syndrome, U1-70k, SmD, Presenilin, Amyloid precursor protein

## Abstract

**Background:**

We recently identified U1 small nuclear ribonucleoprotein (snRNP) tangle-like aggregates and RNA splicing abnormalities in sporadic Alzheimer’s disease (AD). However little is known about snRNP biology in early onset AD due to autosomal dominant genetic mutations or trisomy 21 in Down syndrome. Therefore we investigated snRNP biochemical and pathologic features in these disorders.

**Findings:**

We performed quantitative proteomics and immunohistochemistry in postmortem brain from genetic AD cases. Electron microscopy was used to characterize ultrastructural features of pathologic aggregates. U1-70k and other snRNPs were biochemically enriched in the insoluble fraction of human brain from subjects with presenilin 1 (PS1) mutations. Aggregates of U1 snRNP-immunoreactivity formed cytoplasmic tangle-like structures in cortex of AD subjects with PS1 and amyloid precursor protein (APP) mutations as well as trisomy 21. Ultrastructural analysis with electron microscopy in an APP mutation case demonstrated snRNP immunogold labeling of paired helical filaments (PHF).

**Conclusions:**

These studies identify U1 snRNP pathologic changes in brain of early onset genetic forms of AD. Since dominant genetic mutations and trisomy 21 result in dysfunctional amyloid processing, the findings suggest that aberrant β-amyloid processing may influence U1 snRNP aggregate formation.

## Findings

With age as the greatest risk factor, AD represents a growing challenge in our aging population. AD research over the past few decades has focused on cholinergic dysfunction [1] and its main pathologic constituents: hyperphosphorylated tau and β-amyloid [2]. Thus far, clinical trials of disease modifying therapies targeting these classic markers have been disappointing, and only modest symptomatic therapies are currently available for patients. Further understanding of AD pathogenesis is needed in order to develop viable therapies.

We recently identified enrichment of U1 snRNPs, ribonucleic acid (RNA) processing components, in the insoluble protein fraction in sporadic AD using an unbiased quantitative proteomics approach [3]. RNA splicing is an essential cellular process for converting precursor messenger ribonucleic acid (pre-mRNA) into mature mRNA for use in protein translation [4-6]. RNA processing allows for multiple mRNA and protein isoforms to be created from the same transcript thus increasing genetic heterogeneity. Changes in alternative splicing have been associated with neurodegeneration [7] including AD [8,9].

In our studies, proteomic alterations were validated with immunoblotting and immunohistochemistry that showed accumulation of insoluble U1 snRNP tangle-like structures which partially localized with phospho-tau positive neurofibrillary tangles in prodromal and early stage sporadic AD [3]. Furthermore, RNA sequencing techniques demonstrated widespread RNA splicing dysfunction, including effects on several AD associated genes such as bridging integrator 1 (BIN1) [10], clusterin [11] and presenilin 1 (PS1) [12]. It remains unknown whether the U1 snRNP pathological aggregates are present in genetic forms of AD (PS1 and APP mutations or trisomy 21), but such an association would support a link between U1 snRNP abnormalities and aberrant β-amyloid processing mechanisms in AD.

**Figure 1:**
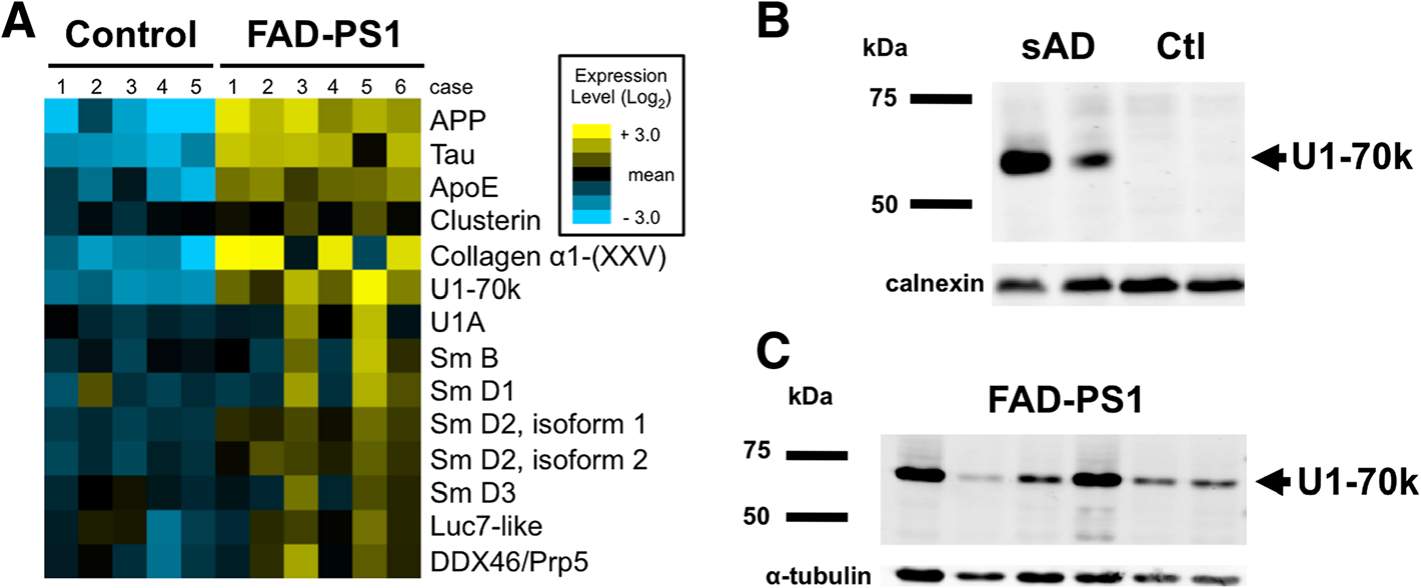
**U1 snRNPs enriched in PS1 insoluble proteome. (A)** Heat map showing quantitative proteomics of frontal cortex from 5 pathology free controls and 6 carriers of pathogenic PS1 mutations (insoluble fraction) demonstrated enrichment (yellow color) of RNA splicing factors and other AD associated proteins in PS1 mutation carriers (FAD-PS1). Log2 of mean of the extracted ion intensities (XIC; normalized protein intensities based on raw signal to noise ratio; Additional file 1: Table S1) in the insoluble fraction from individual cases are shown. **(B)** Western blot showing insoluble U1-70k in two sporadic (sAD) and two control cases with calnexin loading control. **(C)** Western blot showing insoluble U1-70k in 6 PS1 mutation carriers with α-tubulin as loading control.

Since we recently identified U1 snRNP components in the insoluble fraction in sporadic AD [3], we first wanted to determine whether U1 snRNPs were also localized to the insoluble fraction in familial AD (FAD) cases. We therefore compared the insoluble proteome from postmortem frontal cortex of 5 pathology free controls and 6 carriers of pathogenic PS1 mutations (Table 1) with mass spectrometry followed by quantitative proteomic analysis. In addition to the expected AD markers (tau, β-amyloid and apolipoprotein E) and more recently AD-associated collagen type XXV [13], the heat map of extracted ion intensities (XIC, normalized protein intensities based on raw signal to noise ratio) [14,15] demonstrated significant enrichment of U1-70k and Sm-D2 in the FAD insoluble fraction (Figure 1). Sm-B, Sm-D1, and other RNA processing components were also enriched but did not meet statistical significance (p < 0.05; Additional file 1: Table S1). Of the many snRNPs in the brain, the U1 associated snRNPs in Figure 1 were the only ones sequenced in the FAD insoluble fractions. Sm-B and Sm-D isoforms are not exclusive to the U1 snRNP, but thus far we do not have evidence for the involvement of other U complexes in this pathologic process [3]. We also compared the enrichment of snRNPs (Additional file 1: Table S1) in the insoluble fraction for FAD (Additional file 1: Table S1) with our previous findings in sporadic AD cases [3]. U1-70k remained highly enriched in both datasets and, as seen previously in sporadic AD cases [3], protein blotting confirmed U1-70k enrichment in the PS1 insoluble preparation (Figure 1).

**Table 1:** Demographics

**Control**	**PMI**	**Age of death**	**ApoE**	**Gender**	**BRAAK**	**Age onset**	**Mutation**
BLSA-1313	n/a	92	3/3	f	II		
BLSA-1471	n/a	87	2/3	m	II		
BLSA-1517	16	71	4/4	f	II		
BLSA-2027	7	86	2/3	m	IV		
BLSA-2066	17	95	2/3	m	III		
**AD (PS-1):**							
E10-110	42	47	3/3	m	VI	30	M146V
UW-24	3.5	46	n/a	m	VI	39	G209V
UW-238	<24	51	n/a	f	V	41	G209V
UW-301	7	37	n/a	m	VI	31	I143T
UW-304	<24	58	n/a	m	V	48	G209V
UW-328	17.5	44	n/a	f	VI	38	A260V
**AD (APP):**							
E12-24	5.5	54	3/3	m	VI	45	V7171I
UW-16514	n/a	72	n/a	f	VI	62	E693G
**Down:**							
1	20	40	n/a	m	n/a		
2	8	44	n/a	f	n/a		
3	3	46	n/a	m	n/a		
4	5	47	n/a	f	n/a		
5	4.5	59	n/a	f	n/a		
6	15	60	n/a	f	n/a		

*n/a* not available; *BLSA* Baltimore longitudinal study of aging; *E* Emory; *UW* University of Washington; Down syndrome cases from Rush University.

**Figure 2:**
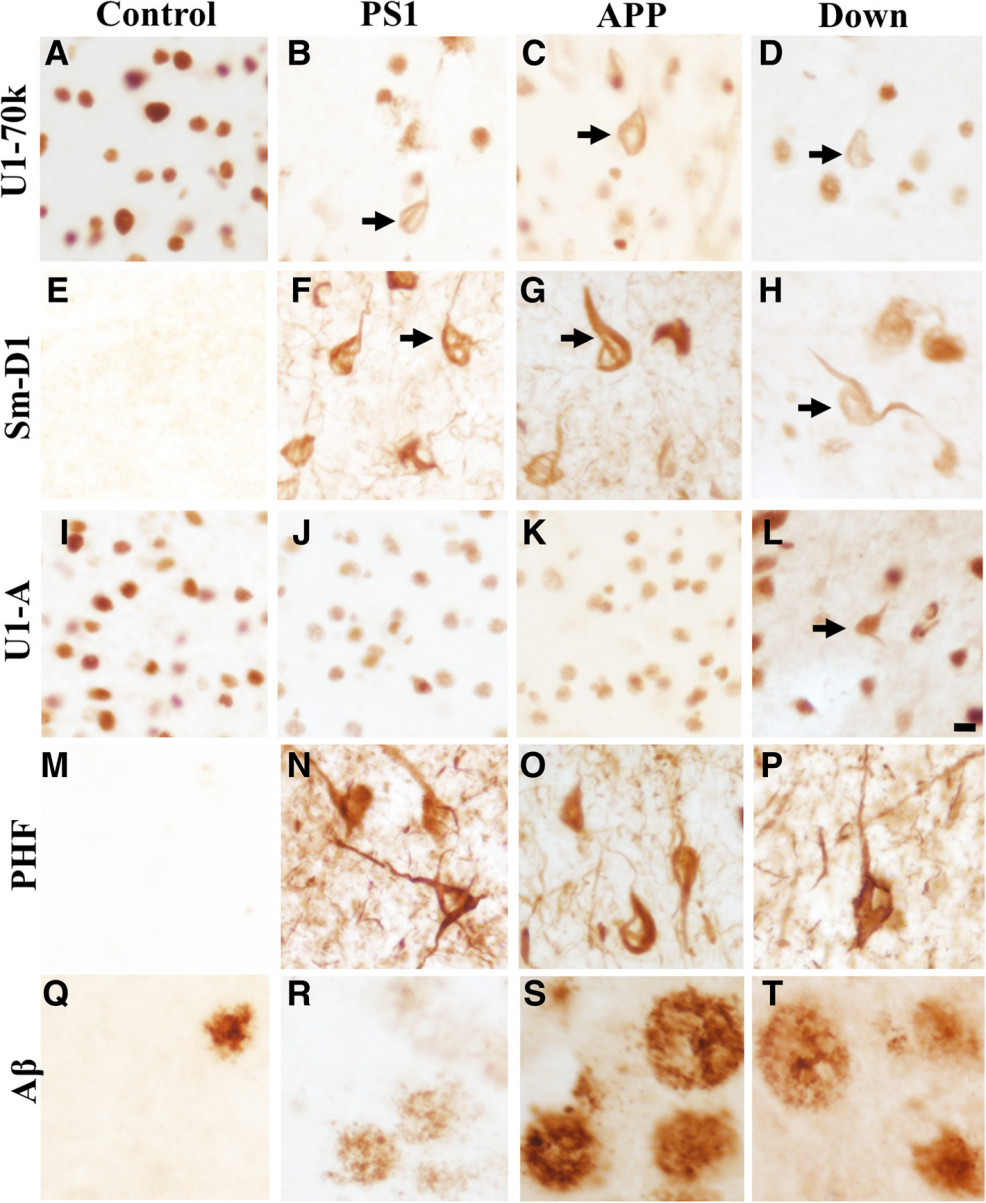
**Immunohistochemistry of U1 snRNPs in FAD and Down syndrome.** DAB immunohistochemistry staining of postmortem human frontal cortex (50 μm free floating sections) from control, PS1 mutation carrier, APP mutation carrier, and Down syndrome patient with **(A-D)** U1-70k, **(E-H)** Sm-D1, and **(I-L)** U1-A. Black arrows designate U1 snRNP tangle-like aggregates. **(M-P)** PHF and **(Q-T)** β-amyloid provided as reference for other AD pathologies. Representative sections shown. Scale bar = 10 μm.

To examine early onset genetic AD brain tissues for evidence of U1 snRNP cytoplasmic aggregates similar to those seen in sporadic AD cases [3], we performed immunohistochemistry of fixed human frontal cortex to localize U1 snRNP in human PS1 and APP pathogenic mutation carriers. We also examined Down syndrome (DS, trisomy 21) cases as patients with DS have an extra copy of amyloid precursor protein and invariably develop Alzheimer’s disease later in life. U1-70k-labeled neurofibrillary structures were observed in the cytoplasm of cortical neurons in most genetic AD cases (PS1, 5/8; APP, 2/2; DS, 5/6; Figure 2). Although Sm-D1 only showed a trend for biochemical enrichment in the FAD insoluble proteome (Figure 1; Additional file 1: Table S1), Sm-D1 immunoreactivity was strongly associated with tangle-like structures in all cases (PS1-6/6, APP-2/2, Down 6/6) (Figure 2). In contrast to our observations in sporadic AD cases [3], U1-A maintained a normal nuclear distribution in genetic AD cases with only rare U1-A tangle structures observed in some Down syndrome cases. β-amyloid positive plaques and hyperphosphorylated tau positive neurofibrillary tangles are shown for reference.

**Figure 3:**
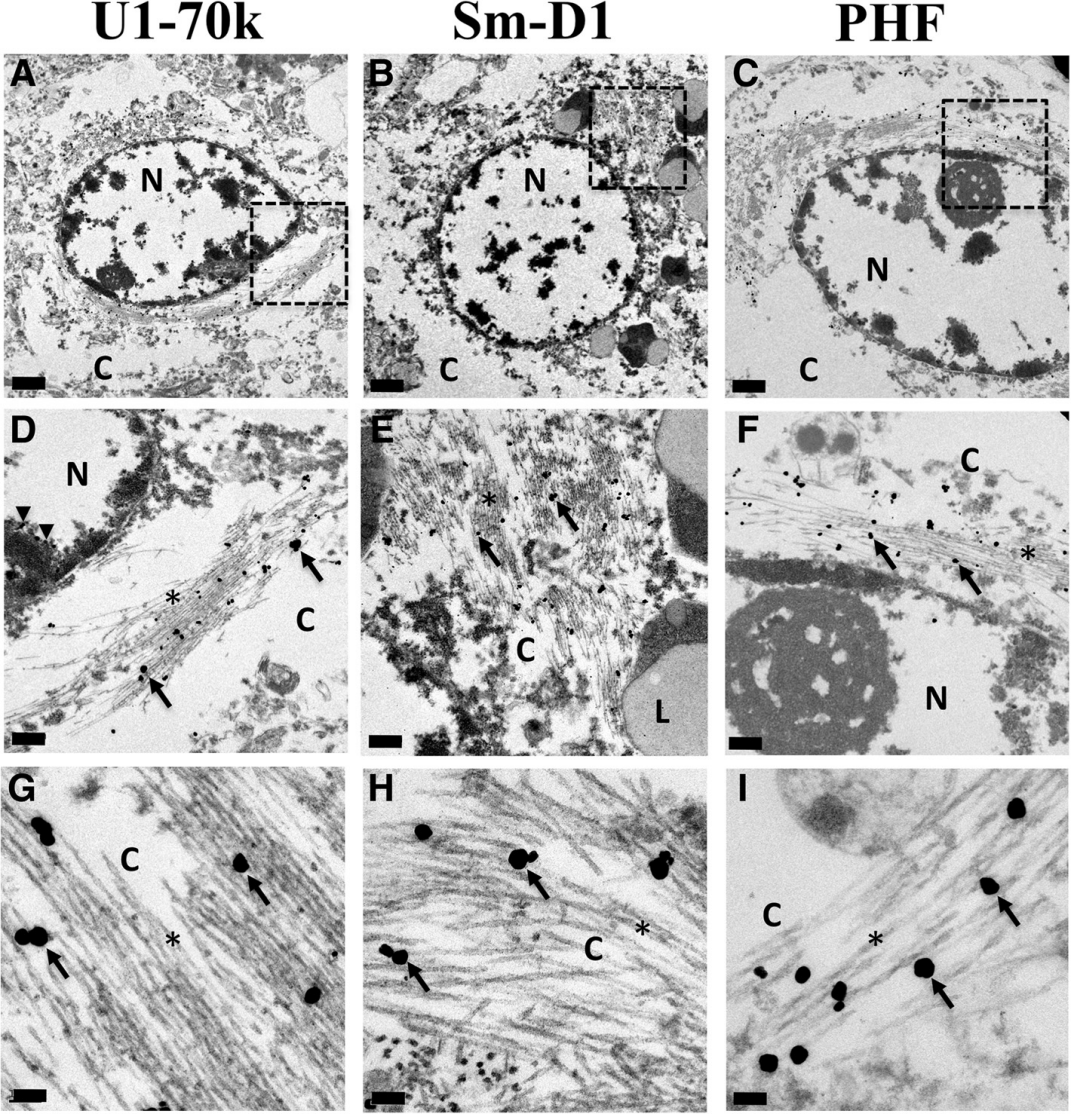
**U1-70k and Sm-D1 immunogold labeling of paired helical filaments in APP mutation carrier.** Transmission electron microscopy was performed on adjacent 50 μm vibratome free-floating sections from an APP mutation carrier following immunogold labeling with **(A, D, G)** U1-70k, **(B, E, H)** Sm-D1 and **(C, F, I)** PHF. Silver enhancement was utilized to label ultrastructural features. Gold particles specifically labeled filamentous structures with all 3 antibodies. N-nuclei, C-cytoplasm, L-lipofuscin granules, black arrows point to immuno-gold particles **(D-I)**, black arrowheads point to nuclear U1-70k **(D)**. Contents of dashed box in **A-C** presented in **D-F**, respectively. Scale bar **(A-C)** 1 μm, **(D-F)** 0.2 μm, **(G-I)** 0.1 μm.

The formation of PHF in AD has been recognized for decades [16-18], and we previously employed dual immunofluorescence microscopy to demonstrate overlap of U1-70k and tau-positive tangles [3]. We sought to obtain more precise definition of the localization of snRNP aggregates in FAD by immunoelectron microscopy. We performed U1-70k and Sm-D1 immunogold staining followed by silver enhancement and transmission electron microscopy in frontal cortex from an APP mutation carrier (Figure 3). In addition to the expected nuclear localization, U1-70k also labeled cytoplasmic fibrils, and staining of adjacent sections demonstrated Sm-D1 and AT8 labeling of morphologically similar structures with characteristic periodicity of ~80 nm. These findings confirm a close association between the snRNP U1-70k and Sm-D1 and pathological aggregates of tau in PHF. We also examined U1-70k immunoelectron microscopy in a PSEN1 mutation carrier (E10-110), however the immunogold staining did not work well and yielded images of poor quality. We were otherwise limited by tissue availability and quality for other FAD cases and did not have sufficient tissues to perform EM in the Down syndrome cases. Because of the pathologic similarities observed in the immunohistochemistry, we did not feel that pursuing more immunoelectron microscopy experiments would provide additional strength to the study.

## Discussion

The pathologic aggregation of proteins is a hallmark of neurodegeneration, and further characterization of these components may provide insights into disease mechanisms. Our recent discovery of U1 snRNP aggregates and reduced RNA splicing efficiency in sporadic AD brains suggests a potential novel mechanism of disease pathogenesis [3]. Our new findings support and extend our previous work by demonstrating the aggregation of U1 snRNPs in cortex of individuals with early onset genetic forms of AD. These findings suggest that snRNP aggregation is involved in the development of AD in cases that are caused by genetically-defined abnormalities in amyloid processing.

How could dysfunctional amyloid processing lead to snRNP aggregation? The common thread between all three genetic subtypes studied in this report (PS1 and APP mutations and trisomy 21) is the relative overproduction of toxic β-amyloid either through altered APP cleavage or APP over-expression. The simplest explanation is that β-amyloid, through a toxic gain of function, drives a series of events leading to U1 snRNP aggregation, similar to the downstream effects of β-amyloid on tau aggregation.

We have demonstrated a close relationship between U1 snRNP aggregates and tau positive neurofibrillary tangles through dual-label immunofluorescence [3] and immunoelectron microscopy (Figure 3). Although snRNPs are mainly found in the nucleus, they are first translated in the cytoplasm and likely rely on cytoskeletal elements including microtubules to reach the nuclear envelope thereby placing tau and splicing components in close association [19]. In addition, tau and various components of the spliceosome undergo phosphorylation [20] perhaps leading to coincidental localization in the course of pathological post-translational modification. The temporal sequence of tau and snRNP aggregate formation (i.e., which pathology occurs first) or whether the different pathologies form independently, remain unknown. In our recent publication [3], we tried to determine if U1 snRNPs aggregated before tau. We studied snRNP aggregates in sporadic AD cases across multiple brain regions and Braak stages. Based on these studies, we did not appreciate robust appearance of snRNP pathologic aggregates in brain regions devoid of tau pathology [3]. We also observed examples of discordant distributions of snRNP aggregates and tau tangles in certain brain regions, like the hippocampus, suggesting the possibility that these are independent processes. Unfortunately, due to the limited availability of tissues and regions with the rare early onset genetic cases, we were not able to evaluate these issues in the current study. Further studies are needed to clarify the association between neurofibrillary tangles and snRNP aggregates in AD.

A significant limitation for proteomics discovery and validation is the availability of antibodies that will work in multiple applications (IHC, protein blotting, etc.). This can be especially challenging when using tissues that may have been fixed for different lengths of time (i.e. tissues with longer fixation can destroy antibody epitopes). For this study, we focused on U1-70k not only because it was the snRNP with high insoluble enrichment in sporadic AD [3] and the snRNP with the greatest fold change in the FAD cases (Additional file 1: Table S1), but also because the U1-70k antibody worked well in immunohistochemistry, immunoelectron microscopy and protein blotting. Although only showing a trend for enrichment, we also focused on Sm-D1 because we found an ideal Sm-D1 antibody that labeled snRNP pathologic aggregates. We attempted to immuno-stain for other enriched snRNPs but were never confident the antibodies (Additional file 2: Table S2) were working consistently. Additional reagents will need to be generated to further examine these other snRNPs.

Other limitations of this study include the relatively small sample size for APP, PS1, and DS cases, factors related to working with postmortem tissues. For example, our mass spectrometric analysis focused on PS1 mutation cases, so differences in APP and DS cases may have been missed. Nevertheless, we observed many similarities in U1 snRNP abnormalities among sporadic and genetic forms of AD. Conversely, in contrast to our findings in sporadic AD cases [3], there appears to be a relative paucity of U1-A pathology in FAD cases. This may represent a key difference between sporadic AD and FAD, but understanding the significance of this observation will require further study. Overall, the results presented in this study significantly extends our previous work in sporadic AD and suggest that U1 snRNP aggregates may play a role in the pathogenesis of all forms of AD.

In summary, we have identified insoluble U1 snRNP aggregates in early onset genetic forms of AD and further characterized the ultrastructural localization of snRNP accumulations. These results suggest that aberrant amyloid processing may directly or indirectly influences snRNP localization and solubility. Further understanding the development of U1 snRNP pathology and contribution of RNA splicing dysfunction in AD may prove critical in our understanding of AD pathogenesis and the identification of novel therapeutic approaches.

## Methods

### Antibodies

A polyclonal rabbit U1-70K antibody was developed and purified using synthetic peptides [3]. Commercial primary antibodies used include: tau (AT8, Pierce-MN1020, Thermo Fisher Scientific, Rockford, IL; 0.1 μg/ml), SmD1 (50940, Abcam; 0.125 μg/ml), U1A (WH0006626M1, Sigma), and β-amyloid (in-house monoclonal which recognizes amino acids 1–16 of β-amyloid; clone 87-5H8; used at 1:1000). Commercial secondary antibodies include biotinylated goat anti-mouse (BA-9200, Vector Labs), biotinylated goat anti-rabbit (BA-1000, Vector Labs), ultra small immunogold goat anti-rabbit (800.011, Aurion), and ultra small immunogold anti-mouse (800.022, Aurion).

### Human tissues

Fresh frozen tissues for preparing brain homogenates and formaldehyde or formalin fixed tissues for immunohistochemistry staining and electron microscopy was obtained from the Emory Alzheimer’s Disease Research Center Neuropathology Core, Atlanta, Georgia, and University of Washington Alzheimer’s Disease Research Center Neuropathology Core, Seattle, Washington. The 5 pathology free controls were obtained from the Johns Hopkins University Brain Resource Center and Baltimore Longitudinal Study of Aging (BLSA) [21]. The Down syndrome cases were obtained from Rush University Medical Center, Chicago, IL.

### Mass spectrometry

Fifty micrograms of protein from the insoluble pellet of 5 AD pathology free human controls and 6 human presenilin 1 mutation carriers were subject to dual mass-spectrometry analysis followed by quantitative proteomic analysis as previously described [3,15,22].

### Immunohistochemistry

Cryopreserved 50 μm free-floating sections from postmortem human frontal cortex from control, PS1 mutation, APP mutation and DS cases were immunostained and visualized with 3,3-diaminobenzidine solution (DAB, Sigma, D4418) as previously described [3,23].

### Electron microscopy

Electron microscopy was performed as previously described [24,25] with use of a JEOL JEM-1400 transmission electron microscope.

## Abbreviations

AD: Alzheimer’s disease; APP: Amyloid precursor protein; BIN1: Bridging integrator 1; mRNA: Messenger ribonucleic acid; DS: Down syndrome; FAD: Familial Alzheimer’s disease; PHF: Paired helical filament; PS1: Presenilin 1; RNA: Ribonucleic acid; sAD: Sporadic AD; snRNP: Small nuclear ribonucleoprotein; XIC: Extracted ion intensities.

## Competing interests

The authors declare they have no competing interests.

## Authors’ contributions

CMH, NTS, EBD, AIL, and JJL. conceived and supervised the project. HY conducted electron microscopy. MG, EJM and JCT provided human samples. CMH performed all immunohistochemistry. EBD performed proteomic analysis. CMH, NTS, EBD, AIL, and JJL all assisted in writing and editing the paper. EJM edited the manuscript. All authors read and approved the final manuscript.

## Supplementary Material

Additional file 1: Table S1 Extracted ion intensities (XIC) from dual mass spectrometry of insoluble brain fraction from individual cases in 5 control and 6 FAD with PSEN1 mutations (left) and proteomic spectral counts of pooled insoluble fractions from control and sAD cases in our previously published dataset (right) [3]. The average fold change of protein enrichment in FAD and sAD as compared to control within each group is shown. Protein spectral counts are shown for the control/sAD comparison instead of XIC values because the original proteomic sequencing run was not optimized for obtaining XIC values. Click here for file

Additional file 2: Table S2 Antibodies that did not function well in immunohistochemistry or protein blotting to demonstrate snRNP aggregates or specific protein enrichment. Click here for file
