# Supplementary material for: U1 small nuclear ribonucleoproteins (snRNPs) aggregate in Alzheimer’s disease due to autosomal dominant genetic mutations and trisomy 21
Source: Mol Neurodegener. 2014 Apr 28;9:15. doi: 10.1186/1750-1326-9-15 (PMC4022210; doi:10.1186/1750-1326-9-15)
Supplement: Additional file 1: Table S1 — Extracted ion intensities (XIC) from dual mass spectrometry of insoluble brain fraction from individual cases in 5 control and 6 FAD with PSEN1 mutations (left) and proteomic spectral counts of pooled insoluble fractions from control and sAD cases in our previously published dataset (right) [3]. The average fold change of protein enrichment in FAD and sAD as compared to control within each group is shown. Protein spectral counts are shown for the control/sAD comparison instead of XIC values because the original proteomic sequencing run was not optimized for obtaining XIC values. [file 1750-1326-9-15-S1.pdf]

Supplementary Table S1: **Extracted ion intensities (XIC) from dual mass spectrometry of insoluble brain fraction from individual cases in 5 control and 6 FAD with PSEN1 mutations (left) and proteomic spectral counts of pooled insoluble fractions from control and sAD cases in our previously published dataset (right) [3]**. The average fold change of protein enrichment in FAD and sAD as compared to control within each group is shown. Protein spectral counts are shown for the control/sAD comparison instead of XIC values because the original proteomic sequencing run was not optmized for obtaining XIC values.

	**Control**					**PSEN1(FAD)**						**Control vs FAD**	**Control vs FAD**
	B-1313	B-1471	B-1517	B-2027	B-2066	E10-110	W-238	W-24	W-301	W-304	W-328	p-values*	Fold change in FAD
**APP**	19.69	68.73	26.81	16.34	12.98	943.14	652	862.1	426.72	609.31	483.63	0.00008	22.9
**Tau**	206.04	195.09	175.6	134.7	230.24	4347.5	3924	3976	3496.6	976.62	3865.07	0.00025	18.2
**ApoE**	90.31	52.07	128.1	36.5	25.35	417.74	500	263.1	376.57	391.65	521.21	0.00003	6.2
**Clusterin**	112.54	182.89	126.7	191.9	200.28	237.61	205.2	371.6	197.04	407.99	222.67	0.03437	1.7
**Collagen a1-(XXV)**	11.16	6.19	7.97	8.24	3.93	399.83	233.1	24.28	211.62	14.69	186.39	0.02749	23.8
**U1-70k**	2.07	2.43	1.48	1.6	1.49	15.51	9.64	29.33	14.45	50.06	19.13	0.01129	12.7
**U1-A**	11.79	8.13	6.73	8.54	7.56	9.16	8.74	38.31	12.4	56.98	10.07	0.16080	2.6
**Sm B**	17.04	24.03	14.03	26.26	23.97	28.22	15.36	66.3	16.16	136.67	41.01	0.19218	2.4
**Sm D1**	2.99	13.73	4.35	3.58	4.53	3.85	4.4	25.67	4.31	29.77	13.64	0.19406	2.3
**Sm D2, isoform 1**	4.69	5.6	4.38	5.16	4.96	12.17	11.19	15.5	9.68	20.66	13.75	0.00074	2.8
**Sm D2, isoform 2**	2.89	3.95	2.82	4.28	3.6	6.58	11.56	10.23	7.59	13.18	8.87	0.00045	2.8
**Sm D3**	15.87	24.08	27.99	19.03	16.05	19.58	16.25	62.94	16.45	45.37	32.72	0.21857	1.6
**Luc7-like**	5.87	10.32	9.98	2.45	4.26	5.24	11.35	13.6	8.51	20.48	11.03	0.09221	1.8
**DDX46/Prp5**	2.67	3.7	2.19	1.1	2.05	2.57	5.18	13.76	3.33	7.65	4.78	0.07044	2.7

	**Control**		**sAD**		**Control vs sporadic AD**
	Control Pool A	Control Pool B	AD Pool A	AD Pool B	Fold change in sAD**
**APP**	9	31	169	196	9.1
**Tau**	10	11	824	989	86.3
**ApoE**	1	1	49	92	70.5
**Clusterin**	87	78	72	75	0.9
**Collagen a1-(XXV)**	1	0	23	24	47.0
**U1-70k**	2	2	31	39	17.5
**U1-A**	0	1	12	22	34.0
**Sm B**	6	6	15	15	2.5
**Sm D1**	7	2	11	11	2.4
**Sm D2, isoform 1**	10	11	20	14	1.6
**Sm D2, isoform 2**	***	***	***	***	***
**Sm D3**	6	3	5	6	1.2
**Luc7-like**	0	1	5	14	19.0
**DDX46/Prp5**	0	0	9	12	10.5

B: Baltimore Longitudinal Study of Aging; E: Emory; W: University of Washington; FAD: familial Alzheimer's disease; sAD: sporadic late-onset Alzheimer's disease. *p-values calculated using Student's ttest. **Unable to perform statistical analysis for significance comparing control and sAD due to nature of dataset with pooled cases. ***Unique peptides to SmD2, isoform 2 were not sequenced.
