# Supplementary material for: U1 small nuclear ribonucleoproteins (snRNPs) aggregate in Alzheimer’s disease due to autosomal dominant genetic mutations and trisomy 21
Source: Mol Neurodegener. 2014 Apr 28;9:15. doi: 10.1186/1750-1326-9-15 (PMC4022210; doi:10.1186/1750-1326-9-15)
Supplement: Additional file 2: Table S2 — Antibodies that did not function well in immunohistochemistry or protein blotting to demonstrate snRNP aggregates or specific protein enrichment. [file 1750-1326-9-15-S2.pdf]

Supplementary Table S2: Antibodies that did not function well in immunohistochemistry or protein blotting to demonstrate snRNP aggregates or specific protein enrichment.

Antibody	Number	Company
DDX46	Ab72083	Abcam
SNRPB	Ab155026	Abcam
SNRPC	Ab122901	Abcam
SNRPD1	10352-1-AP	Proteintech
U1-A	Ab115527	Abcam
